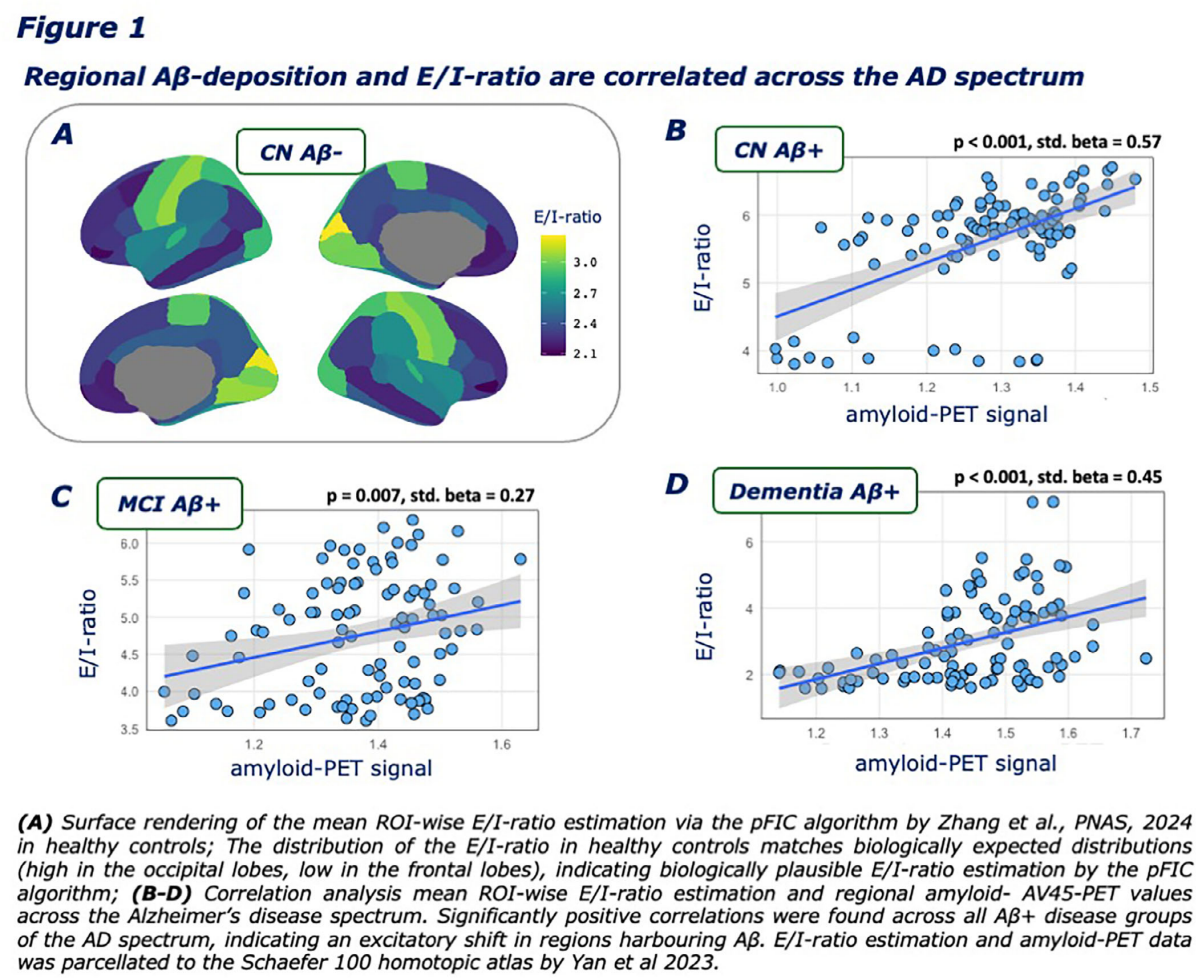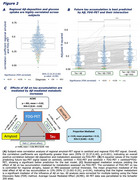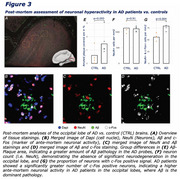# Amyloid‐induced neuronal hyperactivity and ‐metabolism are associated with faster tau accumulation in Alzheimer's Disease

**DOI:** 10.1002/alz70856_099685

**Published:** 2025-12-24

**Authors:** Sebastian Roemer‐Cassiano, Shaoshi Zhang, Lisa Evangelista, Amir Dehsarvi, Madleen Klonowksi, Lukas Frontzkowski, Boris‐Stephan Rauchmann, Anna Steward, Anna Dewenter, Davina Biel, Zeyu Zhu, Fabian Hirsch, Julia Pescoller, Robert Perneczky, Maura Malpetti, Carla Palleis, Johannes Gnörich, Michael Schöll, Martin Dichgans, Sarah Jäkel, Günter U Höglinger, Matthias Brendel, Thomas Yeo, Nicolai Franzmeier

**Affiliations:** ^1^ Department of Neurology, University Hospital, LMU Munich, Munich, Bavaria, Germany; ^2^ Max Planck School of Cognition, Leipzig, Sachsen, Germany; ^3^ Institute for Stroke and Dementia Research (ISD), University Hospital, LMU Munich, Munich, Bavaria, Germany; ^4^ Yong Loo Lin School of Medicine, National University of Singapore, Singapore, Singapore, Singapore; ^5^ Institute for Stroke and Dementia Research, LMU University Hostpital, Munich, Germany; ^6^ Institute for Stroke and Dementia Research (ISD), University Hospital, LMU Munich, Munich, Germany; ^7^ Sheffield Institute for Translational Neuroscience, University of Sheffield, Sheffield, United Kingdom; ^8^ Department of Psychiatry and Psychotherapy, University Hospital, LMU Munich, Munich, Germany; ^9^ German Center for Neurodegenerative Diseases (DZNE), Munich, Germany; ^10^ Department of Neuroradiology, LMU University Hospital, Munich, Germany, Munich, Germany; ^11^ Institute for Stroke and Dementia Research (ISD), LMU University Hospital, Munich, Munich (Bavaria), Germany; ^12^ Ageing Epidemiology (AGE) Research Unit, School of Public Health, Imperial College London, London, United Kingdom; ^13^ Munich Cluster for Systems Neurology (SyNergy), Munich, Germany; ^14^ Department of Clinical Neurosciences and Cambridge University Hospitals NHS Trust, University of Cambridge, Cambridge, United Kingdom; ^15^ Department of Neurology, Klinikum der Ludwig‐Maximilians Universität München, Munich, Bavaria, Germany; ^16^ Department of Nuclear Medicine, University Hospital, LMU Munich, Munich, Germany, Munich, Germany; ^17^ University of Gothenburg, Gothenburg, Västra Götalands län, Sweden; ^18^ Institute for Stroke and Dementia Research (ISD), University Hospital, LMU, Munich, Germany; ^19^ Institute for Stroke and Dementia Research (ISD), LMU University Hospital, Munich, Germany; ^20^ Department of Neurology, Klinikum der Ludwig‐Maximilians Universität München, Munich, Germany; ^21^ Munich Cluster for Systems Neurology (SyNergy), Munich, Bavaria, Germany; ^22^ Institute for Stroke and Dementia Research (ISD), University Hospital, LMU, Munich, Bavaria, Germany; ^23^ Department of Nuclear Medicine, University Hospital, LMU Munich, Munich, Bavaria, Germany; ^24^ Electrical and Computer Engineering, National University of Singapore, Singapore, Singapore; ^25^ University of Gothenburg, The Sahlgrenska Academy, Institute of Neuroscience and Physiology, Psychiatry and Neurochemistry, Gothenburg, Sweden; ^26^ Institute for Stroke and Dementia Research (ISD), LMU University Hospital, LMU, Munich, Bavaria, Germany

## Abstract

**Background:**

The link between amyloid (Aβ) and tau accumulation in Alzheimer's disease (AD) is still unknown, hindering therapeutic efforts to attenuate the Aβ‐tau axis. Preclinical studies demonstrated that Aβ promotes hyperexcitatory neuronal activity and that tau spreads trans‐synaptically in an activity‐dependent manner. We recently showed that tau spreads across connected brain regions, and that Aβ‐related connectivity increases promote tau spreading (Roemer‐Cassiano et al., 2024). Yet, it is unclear whether Aβ‐related hyperconnectivity indeed represents hyperexcitatory neuronal activity. To test this, we combined resting‐state fMRI, FDG‐PET and post‐mortem data, to determine whether Aβ promotes neuronal hyperactivity, thereby driving tau spread in AD.

**Methods:**

We first assessed the effect Aβ on neuronal hyperactivity with a novel algorithm to estimate the excitatory to inhibitory (E/I) ratio applied to resting‐state fMRI in 145 amyloid‐negative controls and 441 amyloid‐positive subjects across the AD spectrum, who also underwent amyloid‐PET. Second, we used glucose metabolism (FDG‐PET) as a marker of neuronal activity in 638 amyloid‐positive AD spectrum patients, with a subset (*n* = 215) of them having tau‐PET at a later timepoint. Lastly, we analysed post‐mortem data of 5 AD patients and 4 controls stained for c‐Fos as a marker of ante‐mortem neuronal activity.

**Results:**

Resting‐state fMRI‐based E/I‐ratio assessment in Aβ‐ controls showed biologically plausible stronger inhibition in association cortices (Figure 1A). In AD, we found an association between higher amyloid‐PET SUVRs and a higher E/I ratio, consistent across diagnostic groups (Figure 1B‐D), indicative of Aβ‐associated hyperexcitatory neuronal activity. Second, we found within individuals, that higher regional amyloid‐PET was linked to higher FDG‐PET (correlation_amyloid‐PET vs. FDG‐PET_: 95% CI [0.37,0.40] *p*‐value <0.001), suggesting higher neuronal activity in Aβ‐harbouring regions (Figure 2A). Similarly, we found post‐mortem elevated neuronal c‐Fos expression in AD brain tissue vs. controls, indicating higher ante‐mortem neuronal activity (Figure 3G). Finally, we found that amyloid‐PET‐based prediction of subject‐level future tau accumulation is improved when including regional FDG‐PET (Figure 2B) and that FDG‐PET‐assessed hypermetabolism mediates subject‐level effects of Aβ on subsequent tau accumulation (Figure 2C).

**Conclusions:**

Aβ promotes an hyper‐excitatory shift in neuronal activity that manifests in glucose hypermetabolism which promotes Aβ‐related tau accumulation. Thus, Aβ‐associated neuronal hyper‐excitability is a potential target for attenuating the Ab‐tau axis in AD.